# Type 1 regulatory T (Tr1) cells: from the bench to the bedside

**DOI:** 10.1186/1479-5876-10-S3-I7

**Published:** 2012-11-28

**Authors:** Silvia Gregori, Rosa Bacchetta, Manuela Battaglia, Maria Grazia Roncarolo

**Affiliations:** 1 San Raffaele Telethon Institute for Gene Therapy (HSR-TIGET), Dept. of Regenerative Medicine, Stem Cells, and Gene Therapy, Milan, Italy; 2 San Raffaele Diabetes Institute (OSR-DRI), Dept. of Immunology, Milan, Italy; 3 Vita-Salute San Raffaele University, Milan, Italy

## Background

Type 1 regulatory T (Tr1) cells are an inducible subset of regulatory T cells that play a pivotal role in promoting and maintaining tolerance. The main mechanisms by which Tr1 cells control immune responses are the secretion of high levels of IL-10, and the killing of myeloid myeloid cells through the release of Granzyme B. To date a defined cell surface signature has not been identified for Tr1 cells and their characterization has thus relied on their unique cytokine production profile. Tr1 cells secrete high levels of IL-10 and minimal amounts of IL-4 and IL-17, which distinguish them from Th2 and Th17 [1]. Furthermore, Tr1 cells secrete low levels of IL-2, and depending on the local cytokine milieu can produce variable levels of IFN-γ. Similar to other T cell subsets, Tr1 cells can transiently express FOXP3 upon activation; however, in Tr1 cells FOXP3 expression is not constitutive and never reaches the high levels characteristic of CD25^+^Foxp3^+^ regulatory T cells**.**

During the last decade, regulatory T cell-based therapies have become an attractive therapeutic option for inducing/restoring tolerance. Several protocols to generate Tr1 cells *in vitro* or to isolate Tr1 cell clones have been developed. We established a reproducible method to generate allo-specific Tr1 cells *in vitro* using recombinant IL-10 or IL-10-derived from tolerogenic dendritic cells (DC).

## Results

Tr1 cells are induced *in vitro* using a new subset of human tolerogenic dendritic cells (DC), termed DC-10, which are present *in vivo* and inducible *in vitro* from monocytes in the presence of IL-10 [2]. Resulting T cells are anergic, secrete significant levels of IL-10, and suppress T cell responses *in vitro* in an IL-10-dependent manner. Adoptive transfer of *ex vivo* induced alloantigen-specific Tr1 cells has proven to be feasible and safe, and can be applied in allogeneic hematopoietic stem cell transplantation. An alternative strategy for the induction of high numbers of human Tr1 cells is lentiviral-mediated gene transfer of human IL-10. Stable ectopic expression of IL-10 can efficiently generate homogeneous populations of Tr1-like cells [3]. These cells display potent suppressive functions both *in vitro* and *in vivo* in xenogeneic graft versus host disease model, while preserving the graft versus leukemia effects.

## Conclusions

Tr1 cells can be induced *in vitro* for Tr1-based cell therapy aimed at restoring peripheral tolerance in immune-mediated diseases.
